# Ferroptosis induced by iron overload promotes fibrosis in ovarian endometriosis and is related to subpopulations of endometrial stromal cells

**DOI:** 10.3389/fphar.2022.930614

**Published:** 2022-09-02

**Authors:** Yanqin Zhang, Xinyu Liu, Mengqi Deng, Chunyu Xu, Yubo Zhang, Di Wu, Fan Tang, Ruiye Yang, Jinwei Miao

**Affiliations:** Department of Gynecologic Oncology, Beijing Obstetrics and Gynecology Hospital, Capital Medical University, Beijing Maternal and Child Health Care Hospital, Beijing, China

**Keywords:** endometriosis (EMs), iron, ferroptosis, fibrosis, stromal cell

## Abstract

Endometriosis (EMs) is defined as the presence of tissue somewhat resembling endometrial glands and stroma outside the uterus; the retrograded endometrium grows in the peritoneal cavity and elicits fibrosis. Ferroptosis is a recently discovered form of programmed cell death, which is iron-dependent. The induction of ferroptosis has been found to participate in fibrosis. However, the relationship between EMs fibrosis and ferroptosis remains unknown. In this study, we confirmed that the iron content in ectopic stromal tissue in ovarian EMs is significantly increased. We explored the role of iron-induced ferroptosis in the pathogenesis of ovarian EMs fibrosis for the first time. We found that ferroptosis in ectopic tissues was significantly enhanced than that in eutopic tissues. Furthermore, we performed *in vivo* drug screening and found that ferroptosis induced by ferric ammonium citrate (FAC) could aggravate fibrosis. To clarify the mechanism of this process, the stromal composition of human uterine endometrium and endometrial tissue was characterized. Fibroblast-specific protein-1 was used for fibroblasts, smooth muscle actin alpha for myofibroblasts, and platelet-derived growth factor receptor beta (CD140b) for mesenchymal stromal cells (MSCs). The results demonstrated that the percentage of myofibroblasts was higher and the portion of MSCs was lower in ectopic endometrial stroma than those in eutopic endometrium. Moreover, the proportion of MSCs decreased significantly and the percentage of myofibroblasts increased considerably after FAC treatment *in vitro*. However, disruption of intracellular iron levels or ferroptosis via chelation of intracellular iron deferoxamine mesylate or ferroptosis inhibitor ferrostatin-1 could reverse this process, indicating that iron-induced ferroptosis plays a vital role in ovarian EMs fibrosis. Considering that iron accumulation can feed the Fenton reaction to generate unquenchable amounts of free radicals, causing ferroptosis and tissue damage and thereby contributing to fibrosis, we validated the underlying mechanism that excess iron can facilitate fibrotic responses. Collectively, these data provide evidence that supernumerary iron is a key regulator in promoting MSCs ferroptosis and inducing ovarian EMs fibrosis.

## Introduction

Endometriosis (EMs) is an estrogen-dependent disease, characterized by the presence of endometrial glands and stroma outside the uterine cavity (Vercellini et al., 2014). It is one of the most common and refractory gynecological disorders, exhibiting malignant features and affecting approximately 5%–10% of fertile women (Zondervan et al., 2018). EMs-related symptoms of fibrotic tissue and pelvic adhesions seriously affect women’s daily life (Matsuzaki and Darcha, 2014). However, the pathogenesis and pathophysiology of EMs fibrosis remain elusive and effective therapies are limited. Innovations may derive from a better understanding of the characteristics of fibrotic lesions at the cellular level.

Fibrosis serves as a complex tissue repair response and a highly dynamic process in chronic injury. Wound healing, tissue remodeling, and repair are protective mechanisms activated in response to stress and injury (Kisseleva and Brenner, 2008). Fibrosis is initiated by functional cell destruction and inflammatory cell activation, in which cell death is driven by various injurious agents and mechanisms (Weiskirchen et al., 2019), leading to local activation of fibroblasts and myofibroblasts, producing the extracellular matrix (ECM). Some researchers have proposed that stroma cells encompass different cell populations. The diversity of a distinct subset of stromal cells has been demonstrated in many multicellular organs, such as the lung (Xie et al., 2018), heart (Bani and Nistri, 2014), and lymph node tissue (Rodda et al., 2018). Subpopulations have been linked to niche and tissue locations, as well as specific functional properties, such as the response to injury by pericyte subsets (Göritz et al., 2011). Considering this, it is likely that endometrial stroma exhibiting the same diversity and complexity of multiple cell subpopulations enables the controlled inflammatory state and scarless repair in the menstrual cycle. Konrad et al. proposed that there are several stromal cell types with different proportions in EMs ectopic and eutopic tissues (Konrad et al., 2018). Taken together, stromal cell subpopulations are vital. However, the functions of these cell subpopulations must be further identified and elucidated.

Iron deposits are considered characteristic of endometrial lesions. Some reports have shown that compared with women without EMs, iron concentrations are significantly higher in the pelvic cavity of patients with EMs (Defrère et al., 2006; Defrère et al., 2008; Polak et al., 2018). Mechanistically, iron catalyzes hydrogen peroxide (H_2_O_2_) in the Fenton reaction, generating highly reactive hydroxyl radicals and higher oxidation states of iron. Oxygen free radicals have been implicated in the pathogenesis of EMs (Foyouzi et al., 2004). The large concentration of iron combined with constant mitochondrial production of H_2_O_2_ renders this reaction an important source of free radicals (Meneghini, 1997). Ferroptosis was first described as a form of iron-dependent programmed cell death in cancer by the Stockwell research group in 2012, in which the hallmark is the contribution of iron to the development of lipid peroxidation and oxidative cell damage (Dixon et al., 2012). Although it is well established that iron overload can trigger ferroptosis, the results of previous studies on ferroptosis resistance and ferroptosis in endometrial lesions are paradoxical (Ng et al., 2020; Li et al., 2021; Li et al., 2022). How iron-induced ferroptosis exerts an influence on EMs fibrosis is not yet elucidated.

## Materials and methods

### Endometrial tissue collection

Fresh endometrial eutopic/ectopic tissues from women with ovarian EMs aged 18–45 years undergoing ovarian cystectomy or laparoscopic surgery were recruited during the late proliferative phase of the menstrual cycle (day 11–13). Samples from 38 women were collected from September 2020 to September 2021 at Beijing Gynecology and Obstetrics Hospital. The diagnosis was confirmed by pathological diagnosis. All pathological samples were obtained from patients who provided their written informed consents. The inclusion criteria were women with normal ovulatory cycles and otherwise normal healthy women who had not received hormonal treatment.

### Isolation and culture of stromal cells

Human ectopic endometrial stromal cells (HEcESCs) were obtained during ovarian cystectomy or laparoscopy in women with ovarian EMs. Human eutopic endometrial stromal cells (HEuESCs) were obtained from the endometrium of the same women with ovarian EMs. Cells were isolated according to methods of primary cell culture for EMs described in our previous articles (Zhang et al., 2021). In brief, the tissues were incubated with 0.25% type IV collagenase–trypsin EDTA solution (Sigma-Aldrich Corporation, St. Louis, MO, United States) at 37°C for 1,015 min, three times. Then, the cells were passed through a 100-μm and 74-μm stainless steel cell filter. Finally, cells were cultured in a maintenance medium composed of 10% dulbecco’s modified eagle medium (DMEM)/F-12 containing 10% fetal bovine serum.

### *In vitro* drug treatment

For *in vitro* drug treatment, 3 × 10^6^ cells were cultured onto a 10 cm well, when cells were grown 70%–80% confluence the drug treatment trials began. To optimize the effects of ferroptosis, we evaluated the cell cytotoxicity in HEcESCs with ferric ammonium citrate (FAC) or erastin. First, cells were seeded in 96-well plates; different doses of FAC (10 mg/L, 20 mg/L, 30 mg/L, 40 mg/L, 50 mg/L, 100 mg/L, 200 mg/L, and 500 mg/L) or different concentrations of erastin (0.5 μM, 1 μM, 1.5 μM, 2 μM, and 2.5 μM) were added and incubated for 24 h. Cell viability was detected using a CCK8 kit. Finally, 50 mg/L of FAC and 1 μM of erastin were used over 24 h in the follow-up experiments. Ferrostatin-1 (Fer-1) is an efficient ferroptosis-specific inhibitor that functions by acting as a classic hydroperoxyl radical scavenger, eliminating the initiating alkoxyl radicals and other rearrangement products produced by ferrous iron from lipid hydroperoxides (Miotto et al., 2020). We used 4 μM Fer-1 to verify the existence of ferroptosis in HEcESCs treated with FAC. At the same time, to verify the role of iron, iron chelator deferoxamine mesylate (DFO) was used to remove iron at a concentration of 100 μM.

### Reagents and antibodies

The following were used in this study: Turnbull blue (Haling, HL80082.4); Iron Assay Kit (Nanjing Jiancheng, A039-2); Prussian blue (Abcam, ab150674); FAC (Sigma Aldrich, A1332); Erastin (Topscience, T1765); Deferoxamine mesylate salt (Sigma Aldrich, D9533); Fer-1 (MedChem, HY-100579); malondialdehyde (MDA; Abcam, ab118970); primary antibodies anti-4HNE (Abcam, ab46545), anti-NOX1 (Abcam, ab55831), anti-COX2 (Abcam, ab23672), anti-fibroblast-specific protein-1 (S100A4; Abcam, ab93283); anti-smooth muscle actin alpha (α-SMA; CST, 19245s); anti-collagen 1 (Abcam, ab34710). antibodies for flow cytometric analysis anti-S100A4 (Biolegend, 370005), anti-CD140b (Biolegend, 323605), anti-α-SMA (Abcam, ab179467), iFluor™488 goat anti-rabbit IgG (H + L), and their respective isotype controls (diluted in 1% serum).

### Iron quantification

Ferrous iron and ferric iron were detected using Turnbull (Fe^2+^) and Prussian (Fe^3+^) blue staining methods, respectively. The total iron content (iron ion and ferritin-bound iron) was assayed with an Iron Assay Kit (Nanjing Jiancheng, A039-2), Turnbull, and Prussian blue staining to detect the localization of iron ion in tissue sections was conducted using freshly prepared 2% potassium ferrocyanide and 2% hydrochloric acid. The tissues were dewaxed and rehydrated with alcohol. Following a 30 min incubation, the sections were rinsed in distilled water and then dehydrated and covered. The stained sections were captured using an optical microscope. Samples were collected, washed with cold PBS, and homogenized in iron assay buffer; then iron reducer was added to the collected supernatant, mixed, and incubated. Finally, the iron probe was added, mixed, and incubated for 1 h, and then was immediately measured using a colorimetric microplate reader at optical density (OD) 593 nm.

### Immunohistochemistry

Samples were fixed with 4% (w/v) neutral paraformaldehyde solution and subsequently embedded in paraffin. Then, serial sections (5 µm) were deparaffinized. The fixed endometrial sections were incubated with anti-4HNE (1:200), anti-NOX1 (1:100), and anti-α-PTGS2 (1:100), followed by HRP-labelled goat anti-rabbit IgG for 1 h at room temperature. Cells and coverslips were mounted using Vectashield with 4′, 6-diamidino-2-phenylindole (DAPI) and analyzed with a fluorescence microscope. The average values were calculated based on the colored area (%) of at least randomly selected magnified images in each sample, which were compared among the groups. Colored areas were quantified using ImageJ software.

### Measurement of MDA

The relative MDA concentration in tissues and cell lysates was determined using a Lipid Peroxidation MDA Assay Kit (Abcam, ab118970), in accordance with the manufacturer’s instructions. Briefly, tissue and cell samples were lysed, and the supernatant (2 ml) was mixed with 2 ml of 0.6% thiobarbituric acid in a 10 ml tube. The tube was heated in a water bath for 60 min, followed by thawing on ice, and the optical density of the solution was further quantified colorimetrically (OD 532 nm).

### Measurement of ROS

The relative ROS concentration in stromal cell lysates was determined using a Cellular ROS Assay Kit (Abcam, ab186029), in accordance with the manufacturer’s instructions. Briefly, plated cells were incubated overnight in a growth medium at 2 × 10^3^ cells/90 µl per well and the corresponding reagent was used for treatment. We added 100 µl/well of ROS Deep Red Working Solution into the cell plate and incubated it in a 37°C, 5% CO_2_ incubator for 30 min. Finally, we monitored a fluorescence increase at Ex/Em = 650/675 nm (cutoff = 665 nm) with bottom read mode and using fluorescent intensity to determine the fold change between control and treated cells.

### Transmission electron microscopy

The supernatant of adherent cells was removed and 2.5% glutaraldehyde was added to fix for 5 min. After centrifugation at 700 rpm for 3 min, the supernatant was discarded. We prepared 1% osmic acid with 0.1 M phosphate buffer (PB) at pH 7.4 was fixed at room temperature for 2 h, rinsed with 0.1 M PB, dehydrated at room temperature with 30%–100% alcohol and 100% acetone, and embedded. After that, the plate was polymerized in an oven at 60°C, and the resin block was cut into 60 nm–80 nm ultra-thin slices.

### Western blotting

Cell samples were incubated with lysis buffer for 30 min on ice. The lysates were then clarified by centrifugation (13,000 rpm) at 4°C for 20 min, and the supernatant was collected for the experiments. Protein concentrations were measured with a BCA kit. Next, samples were fractionated by 10% sodium dodecyl sulfate-polyacrylamide gel electrophoresis (SDS-PAGE) and blotted to polyvinylidene fluoride membranes. Membranes were then incubated for 1 h with 5% skim milk at room temperature. Then, these were probed overnight at 4°C using the following: primary antibodies against α-SMA (1:1,000), collagen 1 (1:1,000), or β-actin. After washing with TBST, membranes were subsequently incubated with secondary antibody at room temperature for 1.5 h and washed with TBST. Finally, suitable secondary antibodies were applied.

### Immunofluorescence staining analysis

Specimen tissues were fixed with 4% polyformaldehyde stationary solution and then soaked in 15% and 30% sucrose solution. Cryosections (5-μm thick) of endometrial tissues (eutopic and ectopic tissues) were incubated with 0.5% Triton X-100 for 15 min at room temperature and then washed in PBS for 3 min, three times. Then, these were aspirated and incubated in 50 μl/well of PBS containing 2% bovine serum albumin for 30 min; the well contents were then aspirated and washed three times with PBS. After incubation for 1 h at 37°C with 30 μl of primary antibody against S100A4 (1:100), α-SMA (1:100), and CD140b (1:100), the cells were washed three times with PBS before incubation with 100 ml of a 1:200 dilution of FITC- or rhodamine-conjugated secondary antibody in PBS containing 2% (w/v) BSA for 1 h at 37°C in the dark. Next, the nuclei were stained with DAPI, and the cover glass was sealed with sealing glue. Finally, the specimens were observed using a confocal laser-scanning microscope.

### Flow cytometric analysis

5 × 10^6^ cultured endometrial HEcESCs and HEuESCs were collected, and cells were successfully extracted to conduct flow cytometric analysis. The cells were washed twice with PBS and treated with Cytofix/Cytoperm Soln Kit (BD, 554714) for 20 min. Then, cells were stained with directly labeled antibodies against S100A4, CD140b, or indirectly labeled antibody α-SMA and second antibody iFluor™488 goat anti-rabbit IgG (H + L) and their respective isotype controls (diluted in 1% serum). After incubation for 30 min at 37°C in the dark, the samples were analyzed using a FACS Calibur flow cytometer (Becton Dickinson), and about 2 × 10^4^ cells were collected to obtain the reported results. The experiment was performed in triplicate.

### Hematoxylin and Eosin staining analysis

Tissues were embedded in paraffin after fixation with 4% paraformaldehyde and dehydrated in ethanol. After paraffin embedding, the sample was cut into 5 μm thick slices. H&E staining was used to confirm the successful establishment of EMs.

### Animal experiments

All protocols of the animal experiments were approved by the Beijing Proteome Research Center (IACUC-20210408-17MO). All 30 BALB/c nude mice (6 weeks, weighing 19 g–21 g) were provided by Viton Levine (Beijing, China) and maintained under specific-pathogen-free conditions. The mice were caged in groups of five for a week before the start of the experiment, with free access to a commercial balanced mouse diet and tap water. The Ethical Review Committee of the Institute approved the experimental protocol.

### Establishment of the EMs mouse model

All 30 BALB/c nude mice were used as recipients of endometrial tissues from patients with EMs. EMs fibrosis was induced using a previously described method with minor modifications (Matsuzaki and Darcha, 2014). The experimental design is summarized in Figure 5A. Each female nude mouse received a single subcutaneous injection of 8–10 endometrial fragments immersed in 200 μl of matrigel on day 0 and then treated with 17-β estradiol (1 mg/kg) *via* intramuscular injection every 5 days (Yuan et al., 2017). Subcutaneous injections were performed using a 1 ml syringe and an 18-gauge needle.

### *In vivo* drug treatment

Drug treatment was initiated on day 14 after endometrial tissue implantation and continued for 2 weeks. Mice were distributed among the treatment groups and divided into six groups. For control mice, corn oil was administered daily, intraperitoneally. FAC (15 mg/kg body weight dissolved in saline, once a day) was administrated intraperitoneally to the second group. The third group received a single intraperitoneal injection of erastin (15 mg/kg, once a day) dissolved in 5% DMSO (Solarbio, D8371) + corn oil (Sigma Aldrich, C8267). The fourth group received intraperitoneal injection of FAC and vehicle. The fifth group received an intraperitoneal injection of FAC and DFO (100 mg/kg). FAC and Fer-1 dissolved in 5% DMSO + corn oil (1 mg/kg, once a day) were administered in the sixth group by intraperitoneal injection. Then, mice were killed by cervical dislocation on day 28 for the collection of endometrial implants. Lesion volume was calculated according to the formula: V = (length × width^2^) × 0.5 (Sun et al., 2012). After being excised and rinsed in phosphate buffer saline, a small portion of the endometrial implants was placed into a 4% paraformaldehyde fixing solution for 24 h and subsequently embedded in paraffin. The remaining portions were quickly used in MDA assay analysis. All experimental procedures were approved by the institutional and local committees on the care and use of animals at the Beijing Proteome Research Center, and all animals received humane care according to the National Institutes of Health (United States) guidelines.

### Statistical analysis

Data were analyzed and graphed using GraphPad Prism. First, the data were tested for normality, and comparisons between two groups were calculated using the Student *t*-test. Groups were compared using one-way and two-way analysis of variance. Differences with a *p*-value < 0.05 were considered significant. The data are expressed as mean ± standard error of the mean unless otherwise specified.

## Results

### Iron level and ferroptosis products increased in ectopic tissues compared with eutopic tissues

**FIGURE 1:**
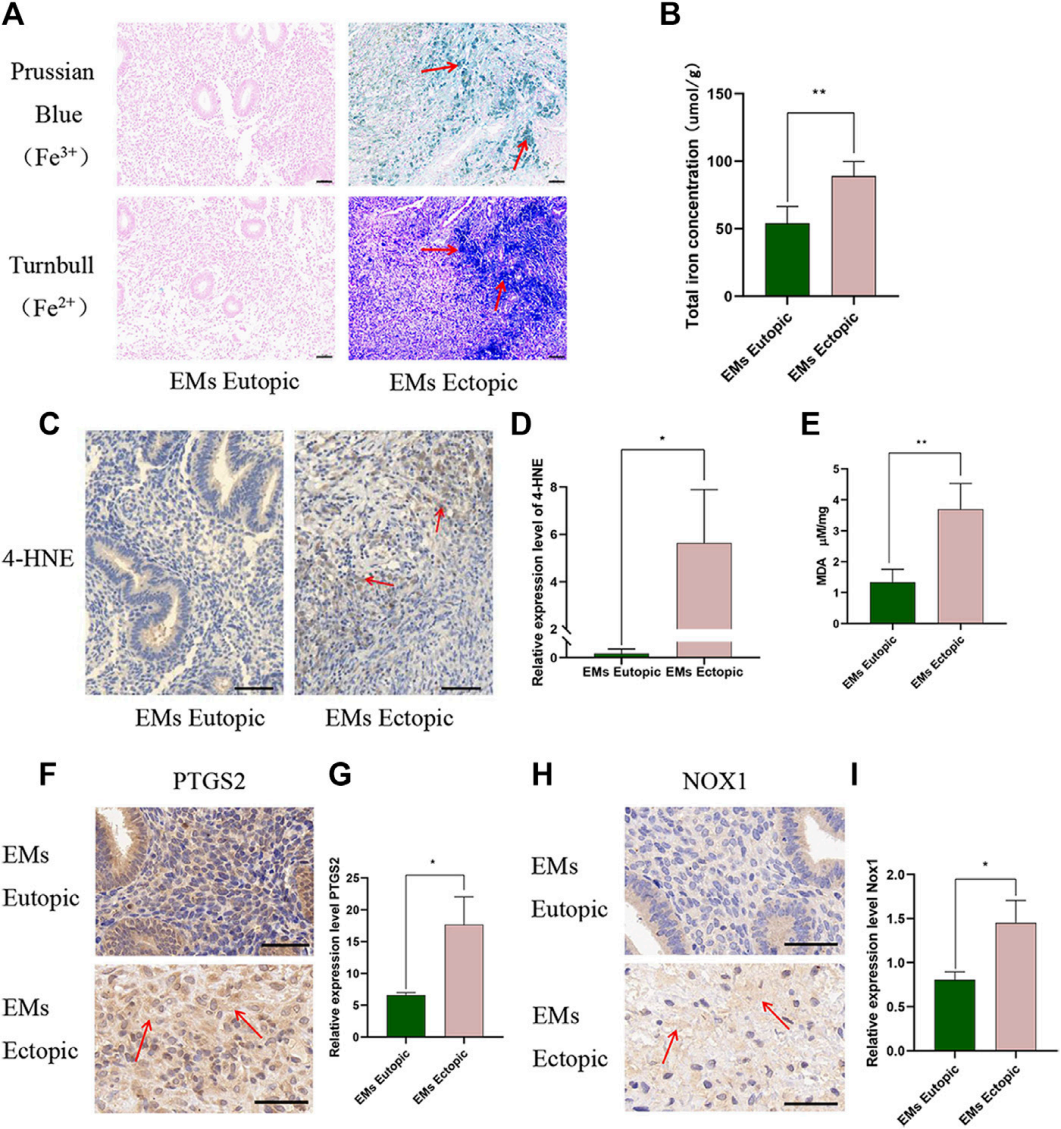
Iron level and ferroptosis products increased in ectopic tissues compared with eutopic tissues. **(A)** Ferric iron (Fe^3+^) and ferrous iron (Fe^2+^) were detected in ectopic tissues compared with eutopic tissues. Ferric iron (Fe^3+^) was detected by Prussian blue, high expression is depicted in green and the ferrous iron (Fe^2+^) was measured by Turnbull high expression is depicted in blue. **(B)** Total iron level (iron ion and ferritin-bound iron) in EMs eutopic and ectopic tissues measured by Iron Assay Kit (Nanjing Jiancheng, A039-2). **(C–D)** Representative images **(C)** and quantitative analyses **(D)** of ectopic and eutopic tissues stained with anti-4-HNE. **(E)** MDA levels in EMs eutopic and ectopic tissues were measured by MDA Assay Kit (ab118970). **(F–G)** Representative images **(F)** and quantitative analyses **(G)** of ectopic and eutopic tissues stained with anti-PTGS2. **(H–I)** Representative images **(H)** and quantitative analyses **(I)** of ectopic and eutopic tissues stained with anti-NOX1. Scale bars: 50 μm. Significance in **(B–F)** was calculated using the Student t-test; *p < 0.05, **p < 0.01, ***p < 0.001.

First, we examined the intensity of iron ion staining with Prussian blue (Fe^3+^) and Turnbull blue (Fe^2+^). Compared with control cases, abnormally hyperintense signals suggestive of Fe^2+^/Fe^3+^ deposits were observed in the ectopic tissues (Figure 1A). Then, the total iron level (iron ion and ferritin-bound iron) was measured using an Iron Assay Kit in ovarian EMs eutopic and ectopic tissues demonstrated that iron in ectopic tissues was notably higher than that in eutopic tissues (Figure 1B, *p* < 0.01). This is consistent with the vast predominance of hemoglobin in ectopic tissue. Iron overload in ectopic tissues has long been recognized as a major trigger of pelvic pain. Currently, ferroptosis can be detected by measuring lipid peroxidation (4-HNE and MDA) and PTGS2 and NOX1 expression (Dalleau et al., 2013; Shimada et al., 2016; Gaschler and Stockwell, 2017). Interestingly, in our study, lipid reactive oxygen species (ROS): 4-NHE (Figures 1C,D, *p* < 0.05), lipid peroxidation product MDA (Figure 1E, *p* < 0.01), and oxidative stress products PTGS2 (Figures 1F,G, *p* < 0.05), and NOX1 (Figures 1H,I, *p* < 0.05) were increased in ectopic tissue. We demonstrated that the expression of fibrosis-associated proteins in ectopic lesions was significantly higher than that in eutopic tissues (Zhang et al., 2021). Therefore, ferroptosis caused by iron accumulation in ovarian EMs may be closely related to ectopic tissue fibrosis.

### In HEcESCs, ferroptosis triggered by iron promotes fibrosis

**FIGURE 2:**
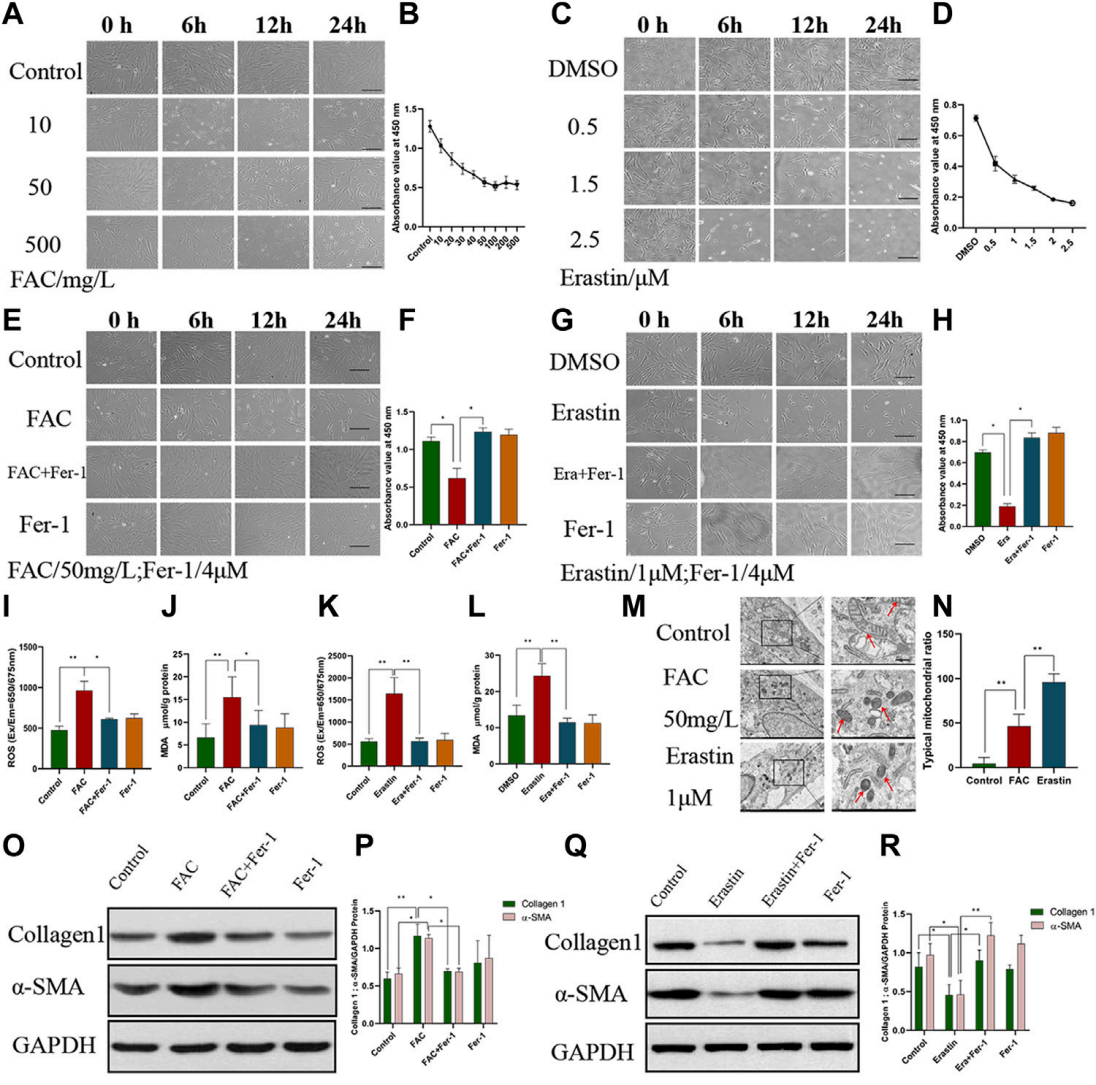
Ferroptosis induced by Ferric ammonium citrate (FAC) could aggravate fibrosis. **(A–B)** Visualization of HEcESCs viability in the different dose of FAC treated groups **(A)** over time and CCK8 assay **(B)** in different treated groups over time. **(C–D)** Visualization of HEcESCs viability in the different dose of erastin treated groups **(C)** over time and CCK8 assay **(D)** in different treated groups over time. With the increasing in drug concentration, all cells in the erastin group tended to be sensitive to ferroptosis, but the cells in the FAC group didn’t exist the same phenomenon after the FAC reached 50 mg/L. **(E–F)** Visualization of HEcESCs viability in FAC and ferroptosis inhibitor Fer-1 **(E)** and CCK8 assay **(F)** in different treated groups over time. **(G–H)** Visualization of HEcESCs viability in erastin and ferroptosis inhibitor Fer-1 **(G)** and CCK8 assay **(H)** in different treated groups over time.The induction of both reagents can be blocked by ferroptosis inhibitor Fer-1. Scar bars: 50 μm. **(I)** The ROS level of HEcESCs treated with FAC and/or Fer-1. **(J)** The MDA level of HEcESCs treated with FAC and/or Fer-1. **(K)** The ROS level of HEcESCs treated with Erastin and/or Fer-1. **(L)** The MDA level of HEcESCs treated with Erastin and/or Fer-1. **(M–N)** Typical mitochondrial picture of ferroptosis under a transmission electron microscope in different treated groups **(M)** and statistical results of typical mitochondrial ratio **(N)**. Only a part of cell mitochondria changed in the FAC-treated group. Scale bars: 500 nm. **(O–P)**. Representative images **(O)** and quantitative analyses **(P)** of the fibrosis-related protein Collagen1 and a-SMA expression levels of HEcESCs in FAC-treated groups. **(Q–R)**. Representative images **(Q)** and quantitative analyses **(R)** of the fibrosis-related protein Collagen1 and a-SMA expression levels of HEcESCs in erastin-treated groups. FAC treatment promoted fibrosis-related protein expressions, while erastin exerted opposite effects. Summary data are presented as the mean ± SEM. Significance in **(F,H–L,N)** was calculated using a one-way analysis of variance; Significance in P and R was calculated using a two-way analysis of variance; *p < 0.05, **p < 0.01, ***p < 0.001.

To clarify the relationship between ferroptosis and ovarian EMs fibrosis, FAC and erastin were used to treat HEcESCs and induce ferroptosis, respectively, and their effect on fibrotic protein α-SMA and collagen 1 were detected by Western blotting. A CCK8 kit was used to establish cell viability after drug treatment. At the same time, ROS and MDA were detected and the transmission electron microscope was used to observe the characteristic change in mitochondria after HEcESCs were treated with FAC and erastin. It is worth noting that with an increasing dose of erastin, the proportion of cells, which initiated the occurrence of ferroptosis, increased gradually until all cells died (Figures 2C,D). However, ferroptosis stopped increasing when the drug concentration reached 50 mg/L in the FAC group (Figures 2A,B). The cell death induced by FAC (Figures 2E,F, *p* < 0.05) and erastin (Figures 2G,H, *p* < 0.05) could be inhibited by the ferroptosis inhibitor Fer-1. Also, marker molecules of ferroptosis ROS and MDA were tested when the cells were treated with FAC (Figures. 2I,J, *p* < 0.01) and erastin (Figures 2K,L, *p* < 0.01), confirming that the ROS and MDA levels could be increased by both of them, and the effect could be reversed by Fer-1. Thus, we validated that ferroptosis can be induced by FAC in HEcESCs. Under a transmission electron microscope, the characteristic change in ferroptosis cells is that mitochondria appear smaller than normal, with increased membrane density. Our results demonstrated that compared with the erastin group, FAC induced the characteristic change of ferroptosis in mitochondria only in a proportion of cells (Figures 2M,N, *p* < 0.01). To unambiguously determine whether upregulated iron concentration contributes to ovarian EMs fibrosis, western blotting was used to detect fibrosis-related proteins. The expression of α-SMA and collagen 1 were found to be upregulated after FAC treatment (Figures 2O,P, *p* < 0.05), whereas erastin exerted opposite effects (Figures 2Q,R, *p* < 0.05). Also, the effects on fibrotic markers of FAC and erastin could be modulated by Fer-1. The above results illustrated that FAC truly induced ferroptosis in some, but not all, HEcESCs and only iron-induced ferroptosis, not erastin-induced ferroptosis, aggravates fibrosis, a very interesting that is worth exploring.

### Heterogeneity of stromal cells in eutopic and ectopic tissues

**FIGURE 3:**
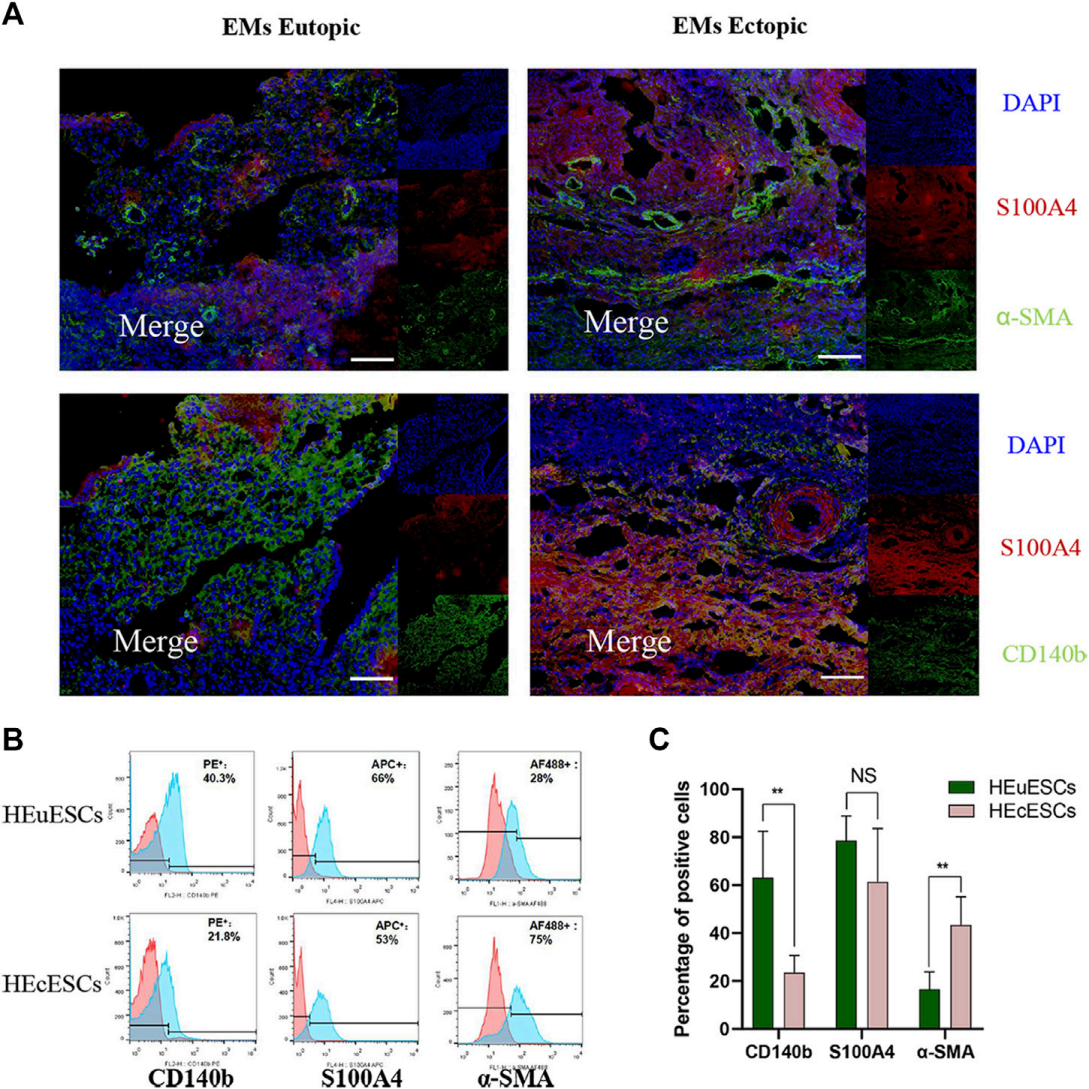
Heterogeneity of stromal cells in eutopic and ectopic tissues. **(A)** Sections of eutopic and ectopic tissues were stained with antibodies against stromal cell markers. CD140b for mesenchymal stromal cells (green), S100A4 for fibroblasts (red), and a-SMA for myofibroblasts (green). There are different kinds of stromal cells with different proportions in ectopic and eutopic tissues of EMs. **(B–C)** Representative FACS analyses **(B)** and statistical results **(C)** of HEuESCs and HEcESCs at early culture passages. The proportion of the CD140b or a-SMA positive cell types in HEuESCs and HEcESCs does differ significantly. Scale bars: 50 μm, Summary data are presented as the mean ± SEM. Significance in **(C)** was calculated using a Student t-test; *p < 0.05, **p < 0.01, ***p < 0.001.

To analyze potential mechanisms in the pathogenesis in ovarian EMs fibrosis, we characterized the stromal composition. In the endometrium, CD140b, S100A4, and α-SMA could be found on immunofluorescence staining, as in ovarian EMs ectopic lesions (Figure 3A). This confirmed that there are different kinds of stromal cells in ectopic and eutopic tissues in ovarian EMs. Sections of eutopic and ectopic tissues were obtained from the same patient with ovarian EMs to conduct the tissues experiment. *Via* fluorescence-activated cell sorting (FACS) analysis, the positive percentage of CD140b, S100A4, and α-SMA was detected (Figure 3B). We were surprised to find that the expression of CD140b in endometrial tissues was lower (Figure 3C, *p* < 0.01) whereas that of α-SMA (Figure 3C, *p* < 0.01) was higher than that in eutopic tissues; no difference was observed in the expression of S100A4, demonstrating the heterogeneity of stromal cells. HEuESCs and HEuESCs were obtained from the same patient with EMs to conduct the cell experiment.

### Iron-induced CD140b-positive cells ferroptosis increased the percentage of α-SMA-positive cells

**FIGURE 4:**
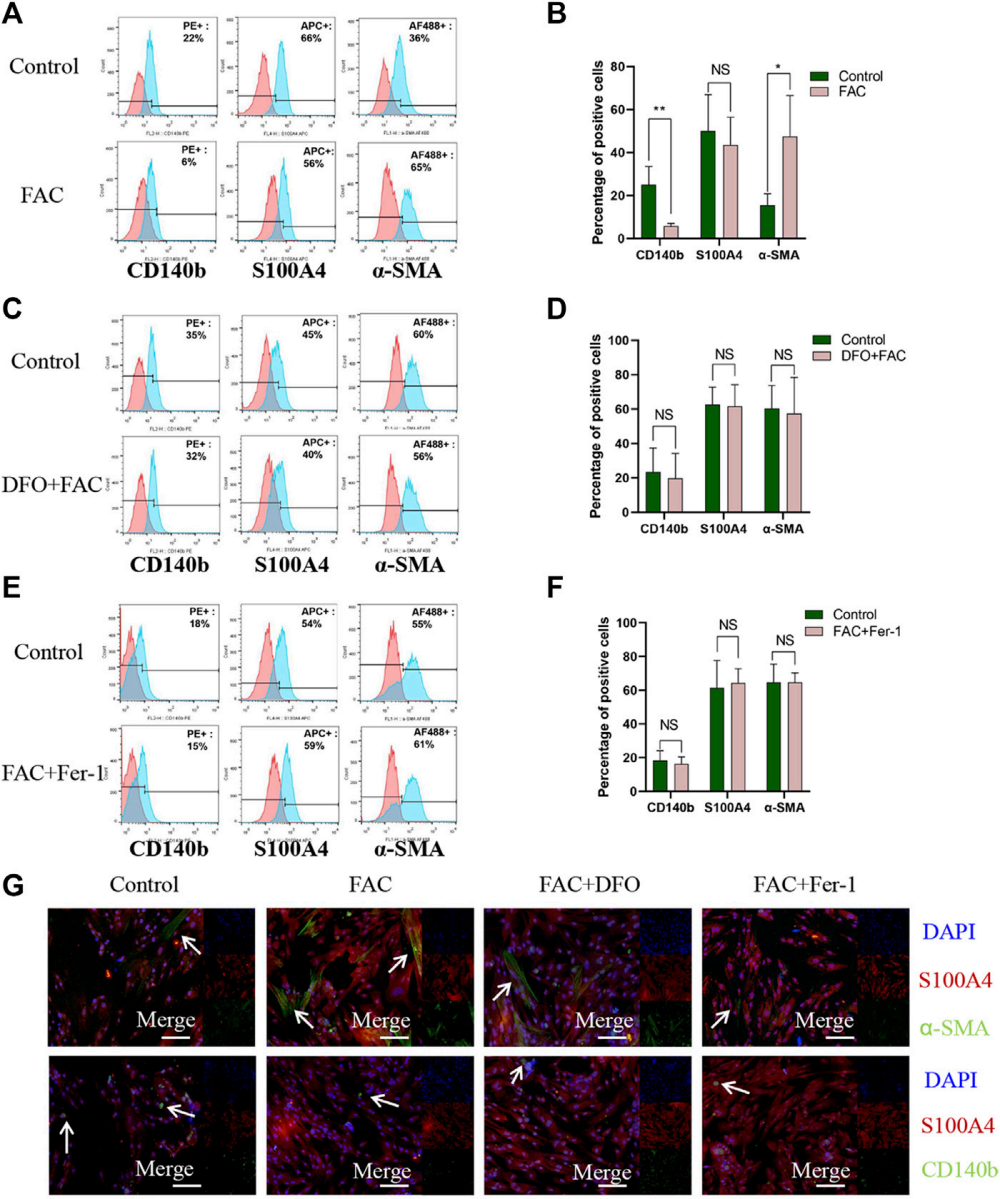
FAC induced ferroptosis of CD140b positive mesenchymal stromal cells and promoted a-SMA positive myofibroblasts activation. **(A–B)** FACS analyses visualization of CD140b, S100A4 and a-SMA positive cell percentage in HEcESCs with FAC treatment **(A)** and statistical results **(B)** in different treated groups. **(C–D)** FACS analyses visualization of CD140b, S100A4 and a-SMA positive cell percentage in HEcESCs with FAC and DFO treatment groups **(C)** and statistical results **(D)** in different treated groups. **(E–F)** FACS analyses visualization of CD140b, S100A4 and a-SMA positive cell percentage in HEcESCs with FAC and Fer-1 treatment groups **(E)** and statistical results **(F)** in different treated groups. FAC can significantly decrease CD140b positive mesenchymal stromal cells percentage and increase a-SMA positive myofibroblasts proportion. **(G)** HEcESCs with different treatment groups were stained with antibodies against stromal cell markers. CD140b for mesenchymal stromal cells (green), S100A4 for fibroblasts (red), and a-SMA for myofibroblasts (green) to further identify the effect of FAC. Scar bar: 50 μm. Summary data are presented as the mean ± SEM. Significance in **(B,D,F)** was calculated using a Student t-test; *p < 0.05, **p < 0.01, ***p < 0.001.

To verify the underlying mechanisms in iron-induced ferroptosis promoting fibrosis and the effects of different cell populations in this process, FAC was used to treat HEcESCs, and then the proportion of CD140b-, S100A4-, and α-SMA-positive cells was determined using FACS analysis and immunofluorescence, respectively. Interestingly, FAC dramatically enhanced the expression of myofibroblast stromal cell marker α-SMA and reduced the expression of CD140b, which is known as the mesenchymal stromal cell marker (Figures 4A,B, *p* < 0.05). The induction can be blocked by iron chelator DFO (Figures 4C,D) and ferroptosis inhibitor Fer-1 (Figures 4E,F). The experimental results were verified again using immunofluorescence (Figure 4G). As we know, FAC can induce ferroptosis in some HEcESCs. Taken together, we verified that in HEcESCs, excess iron induces ferroptosis in mesenchymal stromal cells and ultimately increases the number of α-SMA-positive myofibroblasts, causing the initiation of fibrosis.

### Iron-induced ferroptosis promotes the fibrosis of EMs lesions *in vitro*

**FIGURE 5:**
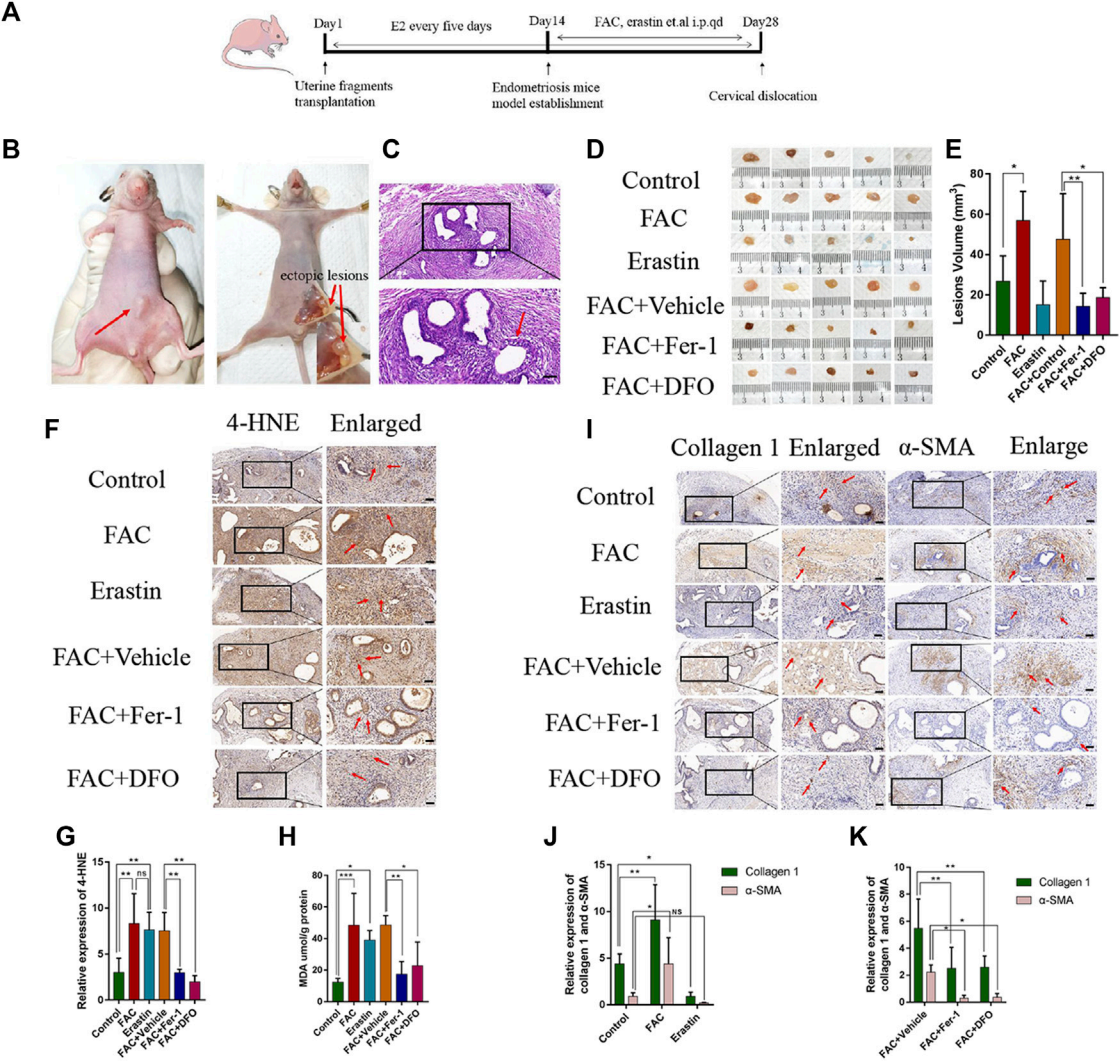
Effects of FAC, erastin and other treatments in the endometriosis mice model. **(A)** Protocol to generate the endometriosis model in Balb/c nude mice. Balb/c nude mice were injected subcutaneously with the endometrial tissues of people with endometriosis and treated with erastin (15 mg/kg/i.p. injection), FAC (15 mg/kg/i.p. injection), vehicle, FAC+Fer-1 (1 mg/kg/i.p. injection), FAC+DFO (100 mg/kg/i.p. injection), FAC+vehicle at day 14 once a day for 2 weeks. Tumor volume was calculated on Day 28 (*n* = 5 mice/group). **(B)** Representative photographs of the endometriosis mice model and visible nodules growth on day 14 after treatment were shown. **(C)** HE staining of endometriotic-like lesions to confirm the success of the endometriosis model, the typical endometrioid gland including highly cylindrical epithelium could be seen. **(D)** Macroscopic aspect of visible lesions on Day 28 was different among groups. The volume of ectopic lesions was enhanced after treatment with 15 mg/kg FAC and tend to reduce after treatment with 15 mg/kg erastin for 2 weeks. In the circumstance of iron overload, Fer-1 and DFO reduce the volume of ectopic lesions. **(E)** Summary data of volume sizes are presented as the mean ± SEM. **(F)** Representative 4-HNE immunohistochemical results of endometriosis ectopic tissues in each group. FAC-treated group and erastin-treated group revealed higher levels of 4-HNE in comparison to other groups. **(G)** Quantitative analysis of 4-HNE in each group. **(H)** MDA content was assayed using a Lipid Peroxidation MDA Assay Kit (Abcam, ab118970). MDA was significantly increased in the FAC and erastin-treated groups. Fer-1 and DFO could reduce the MDA content. **(I)** Representative Collagen1 and α-SMA immunohistochemical results. Fibrosis-related protein immunohistochemical results revealed markedly higher levels of Collagen 1 and α-SMA in the FAC-treated group. In the circumstance of iron overload, the improved effect of iron on endometriosis fibrosis was damaged by treatment with Fer-1 and DFO. **(J)** Quantitative analysis of Collagen 1 and α-SMA. **(K)** Quantitative analysis of Collagen 1 and α-SMA. Scale bars: 50 μm, Significance in **(E,G,H)** was calculated using a one-way analysis of variance; Significance in **(J)** was calculated using a TWO-way analysis of variance;*p < 0.05, **p < 0.01, ***p < 0.001. qd, once a day, i.p., intraperitoneal injection.

To confirm that the mouse model of EMs was successfully established, a nude mouse was randomly selected and killed by cervical dislocation 2 weeks after implantation. Obvious single or multiple nodules could be observed (Figure 5B). H&E staining revealed the successful formation of cystic endometrial lesions with epithelial and stromal cells, and the typical endometrial gland structure, including highly cylindrical epithelium, could be seen (Figure 5C). To further investigate the mechanisms of iron-induced and erastin-induced ferroptosis in EMs fibrosis, six different drug treatments, including vehicle, FAC, erastin, FAC + vehicle, FAC + Fer-1, and FAC + DFO, were administered in mice model. Mice in all groups were killed 2 weeks after drug treatment, and ectopic lesions were harvested. As shown in Figures 5D,E, a different average lesion volume was present among the different treatment groups (*p* < 0.05). Enhanced ectopic lesion size was observed after treatment with FAC, which tended to diminish after treatment with erastin, compared with the control group. In the circumstance of iron overload, Fer-1 and DFO regressed the volume of endometrial implants. This indicates that iron plays an important role in EMs ectopic lesion volume. Additionally, immunohistochemical staining showed that the presence of 4-HNE in the iron and erastin treatment groups was greater than that in the control group (Figures 5F,G, *p* < 0.01), and MDA content showed the same results (Figure 5H), which confirmed that ferroptosis had been initiated. Fer-1 and DFO reduced the content of 4-HNE and MDA in the condition of iron overload, indicating its inhibiting effect on ferroptosis (Figures 5F,H, *p* < 0.05). Immunohistochemical staining also indicated that the expressions of collagen 1 and α-SMA were significantly enhanced in the FAC-treated group compared with those of the erastin and control groups, and under the condition of iron overload, the improved effect of iron on EMs fibrosis was diminished by treatment with Fer-1 and DFO (Figures 5I,J, *p* < 0.05). Although iron and erastin both triggered ferroptosis in our mouse model, iron-induced ferroptosis promoted the initiation of fibrosis and erastin-induced ferroptosis exerted a different influence on EMs fibrosis.

## Discussion

Fibrosis is one of the most common features of endometrial lesions, contributing to tissue adhesions, anatomic distortions, and scarring, and is an important cause of pelvic pain and infertility (Bernacchioni et al., 2021). A study comfirmed that the extent of lesional and cortical fibrosis are correlated with the severity of dysmenorrhea in women with ovarian EMs (Nie et al., 2022). It has been repeatedly demonstrated that endometrial stromal cells that enhance collagen contractility are responsible for increased cellular contractility and collagen production, ultimately leading to fibrosis (Yuge et al., 2007). To explain the pathogenesis of EMs, there are some classic theories like ectopic endometrial implantation and coelomic epithelization (Zondervan et al., 2020). Nowadays, accumulating evidence suggests that immune cells, adhesion molecules, extracellular matrix metalloproteinase, and pro-inflammatory cytokines activate and alter the peritoneal microenvironment, creating the conditions for differentiation, adhesion, proliferation and survival of ectopic endometrial cells (Laganà et al., 2020). However, the further molecular mechanisms of ovarian EMs fibrosis remain elusive. According to Sampson’s hypothesis, the most important process in the development of EMs is menstruation into the peritoneal cavity with repeated retrograde menstrual blood flow (Sampson, 1927). At the same time, periodic bleeding from ovarian ectopic lesions with difficulty in elimination results in local hemoglobin accumulation. Thus, the degradation of hemoglobin in erythrocytes leads to the release of heme; subsequently, the catabolism of heme promotes the formation of many biologically active substances, resulting in excess iron accumulation in the pelvic cavity (Defrère et al., 2008; Polak et al., 2018). In the present work, we confirmed that fibrosis is a feature of ovarian EMs and verified that compared with eutopic endometrial tissues, iron concentration was significantly elevated in ectopic stromal tissues. A growing body of evidence has indicated that impaired iron homeostasis plays a crucial regulatory role in the development of EMs fibrosis. For example, extremely high concentrations of catalytic iron are present in endometrial cysts compared with normal endometrium in the nude mouse model (Van Langendonckt et al., 2004). Mechanistically, it is reported that iron deposition is responsible for the production of ROS and free radicals, such as superoxides, hydrogen peroxide, and hydroxyl radicals, all of which are closely associated with fibrosis and thereby related to infertility (Hayashi et al., 2020).

Ferroptosis is a recently described iron-dependent form of non-apoptotic regulated cell death, characterized by the accumulation of lipid-based ROS and morphological manifestations of smaller mitochondria, increased mitochondrial membrane density, smaller cell volume (Hirschhorn and Stockwell, 2019), and nuclei that maintain their structural integrity, without condensation or chromatin margination (Dixon et al., 2012). Erastin is the prototype ferroptosis inducer that inhibits the function of cystine/glutamate antiporter and reduces the synthesis of glutathione (GSH) by inhibiting the absorption of cystine and decreasing glutathione peroxidase 4 activity (Li et al., 2020). Compared with erastin, iron homeostasis provides an integrated network for determining the sensitivity of ferroptosis via regulators involved in iron metabolisms, such as iron uptake, storage, utilization, and efflux. Mechanistically, it is well established that iron is indispensable in the process of initiating lipid peroxidation in the absence of H_2_O_2_ (Minotti and Aust, 1987; Aaronson et al., 2013). The relationship between ferroptosis induced by iron deposition and fibrosis in EMs is presently unknown. We tested ferroptosis-related markers in endometrial ectopic and eutopic tissue. Our results demonstrated that the expression of molecules related to ferroptosis, such as 4-HNE and MDA, were markedly increased in ectopic stromal tissue in EMs. The obtained results suggested that ferroptosis is enhanced in ectopic stromal tissue of women with EMs fibrosis. Similar to our results, Li G et al. found that endometrial stromal cell ferroptosis promotes angiogenesis in EMs (Li et al., 2022). Consequently, it is speculated that there is an underlying relationship between iron deposition, enhanced ferroptosis, and excessive fibrosis. Therefore, it is worth investigating the mechanisms underlying ferroptosis associated with ovarian EMs fibrosis to improve understanding of the pathogenesis.

To investigate whether iron-induced ferroptosis exerts an influence on ovarian EMs fibrosis and the underlying mechanisms, we conducted *in vivo* and *in vitro* experiments. First, in the presence of an exogenous iron source or erastin, ferroptosis was significantly enhanced in cultured HEcESCs. We found that ferroptosis inducer, erastin, and iron exerted a different influence on the fibrosis-related protein. Compared with the erastin group, FAC induced ferroptosis only in a proportion of HEcESCs, and erastin induced ferroptosis in all HEcESCs. We found that iron-induced ferroptosis enhanced the level of fibrosis-related markers whereas erastin-induced ferroptosis ultimately diminished the level of fibrosis. These findings were consistent with a growing body of evidence that iron concentration is considered a driving factor in the induction of ferroptosis and that iron-dependent ferroptosis is implicated in the pathological process of many fibrosis-related diseases (Fang et al., 2019; Zhang et al., 2020a; Tadokoro et al., 2020). Similarly, it has been observed that ferroptosis induced by erastin attenuates the degree of fibrosis in the liver (Zhang et al., 2018). The above results demonstrated that FAC and erastin can truly induce ferroptosis in HEcESCs and have an opposite influence on ovarian EMs fibrosis. Thus, the question arises, why does iron-induced ferroptosis exacerbate ovarian EMs fibrosis whereas erastin-induced ferroptosis diminishes the level of fibrosis? As reported previously, erastin is the prototype ferroptosis inducer that reduces GSH levels by directly inhibiting the function of cystine/glutamate antiporter (Li et al., 2020). However, intracellular redox-active iron catalyzes the production of lethal lipid ROS and the formation of soluble lipid radicals, either through the Fenton reaction or via the action of iron-dependent oxidases, causing the initiation or propagation of oxidative fragmentation of polyunsaturated fatty acids, enzymatically and non-enzymatically (Hirschhorn and Stockwell, 2019). Combined with the fact that the stromal cellular morphology observed with an optical microscope was inconsistent, a possible explanation is there are different subpopulations of stromal cells, and one such subpopulation is sensitive to ferroptosis induced by FAC, causing initiation and occurrence of fibrosis in other cells. Erastin-induced ferroptosis could reduce the viability of all cells, resulting in a decrease in ovarian EMs fibrosis. Systematic investigation of the mechanism of iron-induced ferroptosis aggravating fibrosis is warranted.

We identified subpopulations of stromal cells in ovarian EMs ectopic endometrial tissue. Histological immunofluorescence analysis and FACS with markers were used for different kinds of cells including S100A4 for fibroblasts, α-SMA for myofibroblasts, as well as CD140b for mesenchymal stromal cells (MSCs). We found a greater proportion of myofibroblasts in the endometrial stroma than in the eutopic endometrial tissue whereas the proportion of CD140b-positive cells was the opposite. After FAC treatment, iron-induced ferroptosis resulted in profound changes in the subpopulation of stromal cells, among which decreased MSCs expressing CD140b and increased myofibroblasts expressing α-SMA were the predominant characteristics. These findings were consistent with previous reports that stromal cells harbor multiple stromal populations (Bani and Nistri, 2014; Rodda et al., 2018; Xie et al., 2018). Furthermore, in research concerning EMs stromal cells, Konrad et al. characterized the existence of stromal cell subpopulations in the endometrium with or without EMs using different markers (Konrad et al., 2018). To explore cellular interactions in the process of endometrial regeneration, Queckbörner used single-cell RNA sequencing and identified 10 different stromal cell subpopulations and two pericyte subsets existing in the stroma, establishing cell cluster diversity and indicating that the endometrial stromal compartment is very complex (Queckbörner et al., 2021). Taken together and considering possible differences between RNA levels and protein levels, the subpopulations and specific functions of EMs stromal cells are worth further study. Myofibroblasts are proposed as the principal source of ECM in fibrosis. Our study revealed vital theoretical evidence for explaining the mechanisms of fibrosis exacerbated by iron-induced ferroptosis in ovarian EMs, namely, ferroptosis induced by iron in stromal cells triggers the activation of fibrosis in ovarian EMs.

To further validate the biological role of iron-induced or erastin-induced ferroptosis *in vivo*, we first established a mouse model of EMs. Ectopic lesions in the experimental group were established successfully, and the results confirmed the aforementioned conclusions that ferroptosis induced by FAC could aggravate fibrosis whereas ferroptosis induced by erastin maybe play a key role in the inhibition of fibrotic protein levels. The inhibitory effect of erastin on EMs lesions was also reported by Li et al. (2021). Yu et al. reported that feeding hepatocyte-specific transferrin knockout (Trf-LKO) mouse with impaired iron metabolism a high-iron diet increased their susceptibility to developing ferroptosis-induced liver fibrosis and Fer-1 could potently reduce liver fibrosis triggered by iron-induced ferroptosis (Yu et al., 2020). Zhang et al. found that inducing hepatic stellate cells ferroptosis could be an effective manner to alleviate murine liver fibrosis (Zhang et al., 2020b). Compared with control mice, the myocardium of mice with heart failure was reported to exhibit obvious fibrosis combined with higher levels of iron and MDA, which indicated that the occurrence of ferroptosis maybe plays a key role in fibrosis (Zheng et al., 2021). Therefore, most studies were consistent with our results confirming the fact that iron-induced ferroptosis is a promoter of fibrosis whereas erastin-induced ferroptosis tends to reduce the level of fibrosis. However, Gong found that erastin-induced ferroptosis can promote fibroblast-to-myofibroblast differentiation whereas ferroptosis inhibitors such as Fer-1 reduce the level of pulmonary fibrosis (Gong et al., 2019). We consider that this difference may be related to the different types of disease models.

Ferroptosis is related to fibrosis in many organs and is reported differently in different studies. For example, Li et al. found that inhibiting ferroptosis is considered as an effective way to reduce radiation-induced lung fibrosis (Li et al., 2019) Wang et al. reported that Fer-1 treatment or knockout of p53 inhibits the occurrence of ferroptosis in HSCs can aggravate liver fibrosis in mice (Wang et al., 2019). With the help of intrinsic iron overloading, activation of ferroptosis may be a vital mechanism for inducing fibrosis. Fang et al. reported that the application of Fer-1 may be a therapeutic approach to diminish myocardial fibrosis caused by iron-induced ferroptosis in mice (Minotti and Aust, 1987). Those authors also identified that iron accumulation in liver tissues could initiate ferroptosis-induced liver fibrosis (Zhang et al., 2018). Notably, a few studies have explored the relationship between ferroptosis and EMs. For example, Ng proposed that abnormal eutopic endometrium in EMs is resistant to ferroptosis, but the mechanism is still not clear (Ng et al., 2020). Li found that erastin can induce ferroptosis and reduce the volume of ectopic endometrial tissues in a mouse model (Zhang et al., 2020a). There is no evidence regarding the relationship between ferroptosis induced by iron and ovarian EMs fibrosis. In our study, we systematically explored the effects of iron-induced ferroptosis on EMs fibrosis and found that ferroptosis induced by excessive iron promotes fibrosis in ovarian EMs. Additionally, we first demonstrated that subpopulations of stromal cells may play a role in this process.

## Conclusion

Taken together, our data demonstrated that endometrial stromal cell ferroptosis is predominantly induced by increased iron concentration, ultimately causing fibrosis. In this process, we verified the existence of a stromal cell subpopulation. Also, CD140b-positive endometrial stromal cells were significantly decreased and α-SMA-positive myofibroblasts were increased with FAC treatment. Additionally, erastin-triggered ferroptosis could alleviate fibrosis. Repeated cell death is a major cause of tissue injury and repair, which is a core of fibrosis. We regard the occurrence of ferroptosis in CD140b-positive cells induced by iron overload in ectopic tissue to be the main factor in ovarian EMs fibrosis. The above results provide new insights into the development of ovarian EMs fibrosis and indicate that the composition of endometrial ectopic cells is very complex. Some limitations exist in our studies; therefore, further research is warranted to clarify and further confirm the endometrial stromal cell comprise subpopulations and the function of these cell populations. Additional investigations are needed to identify which subpopulation of cells, and specific bioactive factors and cytokines, are involved in the occurrence of ferroptosis and activation of fibrosis. Understanding the molecular mechanisms underlying the regulation of iron metabolism during ferroptosis may provide effective strategies for the treatment of ferroptosis-associated ovarian EMs fibrosis. Controlling the occurrence of ferroptosis, in turn, emerges as having fundamental importance and offers novel opportunities for potential therapeutic intervention of ovarian EMs fibrosis.

## Data Availability

The original contributions presented in the study are included in the article/supplementary material, further inquiries can be directed to the corresponding author.

## Abbreviations

EMs, Endometriosis; FAC, Ferric ammonium citrate; S100A4, Fibroblast-specific protein-1; α-SMA, smooth muscle actin alpha; MSCs, mesenchymal stromal cells; CD140b, platelet derived growth factor receptor beta (Pdgfrb); DFO, Desferrioxamine mesylate; Fer-1, ferrostatin-1; ECM, extracellular matrix; H_2_O_2_, hydrogen peroxide; ROS, reactive oxygen species; HEcESCs, human ectopic endometrial stromal cells; HEuESCs, Human eutopic endometrial stromal cells; 4-HNE, 4-Hydroxynonenal; NOX1, NADPH oxidase1; PTGS2, Prostaglandin-endoperoxide synthase 2; FACS, fluorescence activated cell sorting; GSH, Glutathione; MDA, malondialdehyde; EMT, epithelial-mesenchymal transition; FMT, fibroblast-to-myofibroblast transdifferentiation; SMM, smooth muscle metaplasia; PUFA-PLs, polyunsaturated fatty acid phospholipids.
